# The Hair

**Published:** 1892-01

**Authors:** 


### THE HAIR.

Too constant washing of the hair is unnecessary, as well as harmful. Once a week is quite often enough for cleanliness, as well as for maintaining the strength of the hair. The same remark applies to constant brushing, for continual brushing, especially with hard brushes, should be avoided. There is a common notion that greasing the hair is vulgar ; so many other persons fall into the other extreme, and never apply any pomade at all. After the hair has been washed, it is certainly beneficial to apply some form of simple grease or oil. When the head-hair is becoming rapidly thinned, some stimulating material, such as ammonia and cantharides, added to the oil, will increase its good effects.

